# A dataset of the 2023 presidential election in Nigeria

**DOI:** 10.1016/j.dib.2024.110847

**Published:** 2024-08-28

**Authors:** Adenike Tosin Odegbile, Olufemi Moses Oyelami

**Affiliations:** Computer Science Programme, Bowen University, Iwo, Nigeria

**Keywords:** Sentiment Analysis, Deep Learning, Python, Twitter

## Abstract

Nigeria operates under a multi-party system with more than 18 registered political parties. Since the return to democratic rule in 1999, the political scene has been predominantly dominated by two major parties: the People's Democratic Party (PDP) and the All Progressive Congress (APC). Recently, however, emerging parties like The Labour Party (LP) and the New Nigerian People's Party (NNPP) have started gaining traction. Social media has become a pivotal part of modern society. Twitter (now known as X) has emerged as a significant medium for news dissemination, public opinions expression, and emotional responses on various topics. Its ability to allow real-time sharing of views and experiences on current affairs and personal matters has made it a powerful tool in shaping and reflecting public sentiment. The use of Twitter in Nigeria exemplifies its role as a versatile medium for expressing thoughts and feelings, thereby generating a substantial amount of data for sentiment analysis. Deep Learning is a branch of Artificial intelligence that uses multiple layer techniques to extract features from data. It has the capacity to adequately recognize pattern from data to produce insights. There is a dynamic interplay among political developments, social media use, and sentiment analysis using deep learning. This interplay highlights the evolving nature of public discourse and opinion formation in Nigeria. People's opinions about the Nigeria's 2023 Presidential Election were obtained from Twitter using the Twitter API and Python. The dataset contains 364,867 tweets that can be used in predicting the outcome of future elections in Nigeria and for comparing the performances of different models and techniques of sentiment analysis.

Sentiment analysis; Deep learning; Python; Twitter

Specifications Table

**Table:** 

Subject	Deep Learning.
Specific subject area	Data Collection and Analysis of Tweets based on Nigeria's 2023 presidential election
Type of data	Table (CSV)
Data collection	A total of 367,701 tweets based on Nigeria's 2023 presidential election were collected. The data was mined from Twitter using following hashtags #obi, #atiku, #bat, #Obidatti, # Atikulate and #batified from 01/08/2022 to 28/02/2023. The hashtags represent the three major presidential candidates who are Peter Obi, Bola Ahmed Tinubu and Atiku Abubakar. The data was mined using the Twitter API and the data was pre-processed to remove stopwords.
Data source location	Country: Nigeria
Data accessibility	Repository name: Mendeley Data Data identification number: 10.17632/whb6rychpx.2 Direct URL to data: https://data.mendeley.com/datasets/whb6rychpx/2
Related research article	None

## 1 Value of the Data

• This dataset will provide insight into people's sentiment to understand their views towards the 2023 Nigeria presidential election on Twitter.
• This dataset will be valuable to policymakers, politicians and researchers to help them understand people's opinions about Nigeria's 2023 presidential election. The insight from the data can then be used to develop policies that relate to the general public. The insight gained from the data will also help each of the candidates and their respective political parties identify their areas of strengths and weaknesses so as to be able to make amends where necessary for future elections.
• This dataset can help researchers interested in Natural Language Processing to gain insight into people's opinion towards Nigeria's 2023 presidential election and possibly make predictions towards the outcome of the subsequent Nigeria's presidential elections by using the sentiment label and sentiment score to train models and the trained models can be used to make prediction on new or unseen data.
• The dataset will assist the candidates and their political parties to understand the yearnings and needs of the populace so as to be able to care of them.
• The “created” column in the dataset which contains the time and date each tweet was created can be used for time series analysis of the tweets. This can be used for trend identification, anomaly detection and enhanced visualization.
• Lastly, the dataset will serve as text data for comparing the performances of different machine learning algorithms.

## 2 Background

The data was obtained to compare the result of Convolutional Neural Networks, Recurrent Neural Networks and Transformer-Based approaches in sentiment analysis using Nigeria's 2023 presidential election opinion data as a case study.

## 3 Data Description

This dataset contains tweets about the three major presidential candidates of Nigeria's 2023 presidential election. The three major candidates are Abubakar Atiku of The People's Democratic Party (PDP), Bola Ahmed Tinubu (APC) and Peter Obi of the Labour Party (LP). The data mined for each of the major three candidates is stored in the “Raw Data” folder with the names of the candidates while the preprocessed data is in the “Cleaned Data” folder and the “Labelled Data” folder contains the labelled text.

### 3.1 Mined data (Folder: raw data)

The data used in the research was mined from August 2022 to February 2023. The data was extracted based on the three major presidential aspirants of the 2023 Presidential election who are Atiku Abubakar, Bola Ahmed Tinubu and Peter Obi. A total of 224,579 data was mined from Twitter between 01/08/2022 and 28/02/2023 using #obi, #atiku, and #bat hashtags while 140,288 data was mined using #ObiDatti, #Atikulate, #batified. The data of the candidates are stored in the folder named “Raw Data”. The data of each of the candidates was merged and preprocessed. The preprocessed data stored in the “Clean Data” folder while the labelled dataset is stored in the “Labelled Data” folder.

**Fig. 1:**
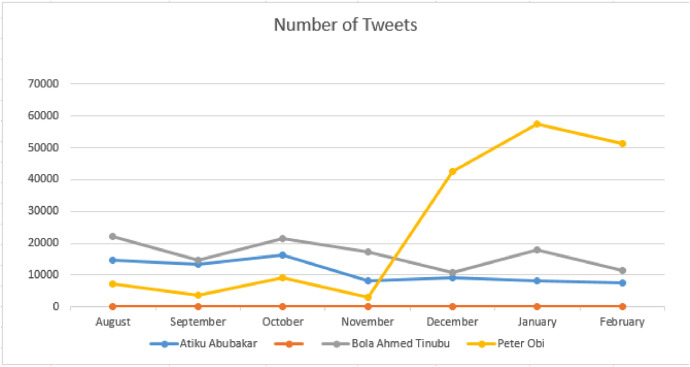
Tweets Discussing Nigeria's 2023 Presidential Candidates from August 2022 to February 2023.

The mined data was stored as a csv file labelled with the names of the candidates. The file contains seven columns. The first column contains the serial number of the data mined starting from 0. The second columns. The second column is the language of the text mined. The languages in the raw dataset include English (en), da (danish), tr (turkish), and und (Undetermined), etc. The third column contains the main text of the tweet. The RT and @ in some of the text signifies that the text was retweeted and the @ points to the original author of the tweet. The fourth column shows the date and time the tweets were created. The fifth column contains the number of times the text was retweeted while the sixth column signifies the number of replies on the tweets and the seventh column shows the number of times the text was quoted by others on Twitter. Table 1 contains numbers of tweets mined for each candidates while Table 2 contains the total number of tweets for all candidates. Table 3 consists of the monthly distribution of tweets mined for all three candidates and Fig. 1 contains a graphical representation of the monthly tweets mined for each candidates.

**Table 1:** Numbers of tweets mined for all candidates.

Feature	Description	Detail
Language	The language of the tweets	English Language
Text	The text contained in the Twitter post	String
Retweets	The number of the times the post was retweeted	163,671,961
Num_replies	The number of replies on each post	43,213
Quotes	The number of times the post was quoted	8734
Created	The date the post was created	Date

**Table 2:** Total number of tweets for all candidates.

Candidate	Hashtags	Number of tweets
Atiku Abubakar	#atiku #Atikulate	76,774 516
Bola Ahmed Tinubu	#bat #batified	112,700 2983
Peter Obi	#obi #ObiDatti	37,979 136,749

**Table 3:** The number of tweets mined for each candidate monthly.

Candidates	August 2022	September 2022	October 2022	November 2022	December 2022	January 2023	February 2023
Atiku Abubakar	14,593	13,286	16,435	8057	9221	8068	7630
Bola Ahmed Tinubu	22,097	14,618	21,401	17,187	10,960	17,859	11,561
Peter Obi	7260	3793	9165	2944	42,711	57,637	51,218

### 3.2 Preprocessed data (Folder: cleaned data)

The data was cleaned and preprocessed to remove stopwords, punctuations, retweet-pattern and emojis. While cleaning the data, non-English text were removed and only English text were retained in the dataset. The initial total number of tweets mined was 367,701 tweets and after cleaning and preprocessing, the data decreased to 325,930.

The preprocessed dataset was saved as a csv file and it contains seven columns. The first column contains the serial number of the preprocessed data. The second column is the language of the text. The third text column contains text without emoji, special characters, duplicates, punctuation and stopwords. The fourth column specifies the number of retweets of a tweet while the fifth column is the number of replies followed by the sixth column that contains the number of times a tweet was quoted and the seventh column contains the date the tweet was made.

### 3.3 Labelled data (Folder: labelled data)

The Labelled data folder contains two csv files which are labelled.csv and cleaned_labelledsentiment.csv. The labelled.csv file contains 325,930 sentiment labelled data while the cleaned_labelledsentiment.csv file contains 117,656 unique sentiment labelled data without duplicates.

**Fig. 2:**
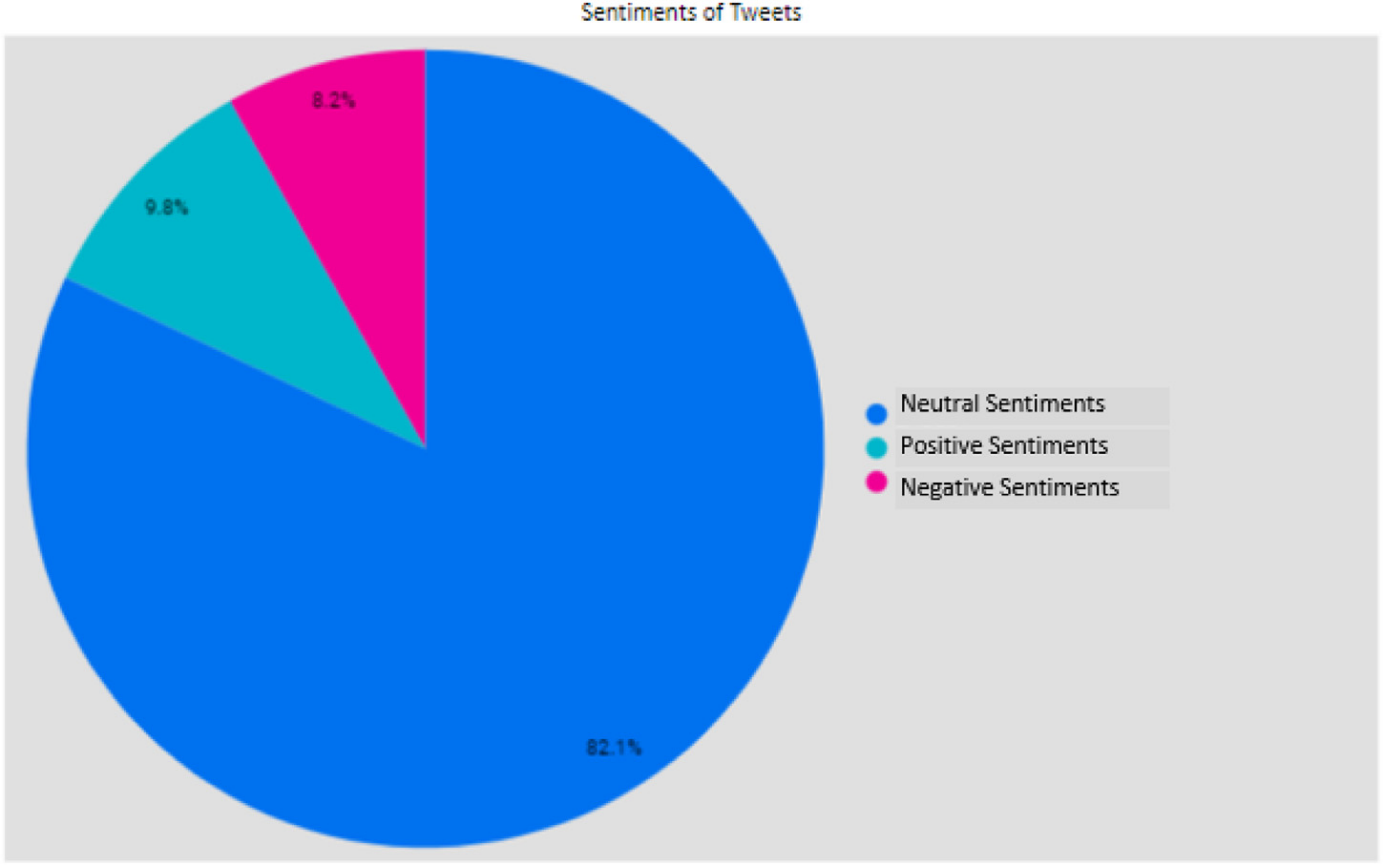
Sentiment count of tweets discussing Nigeria's 2023 presidential candidates from August 2022 to February 2023.

The data was labelled using the cardiffnlp/twitter-roberta-base-sentiment model. The Roberta pre-trained model can handle ambiguity [1]. The model was specifically trained using Twitter data which allows it to handle abbreviations and informal languages. The data is labelled 0, 1 or 2. Label 0 represents negative sentiments, Label 1 represents neutral sentiments and Label 2 represents positive sentiments. The sentiments of the tweets are presented pictorially in Fig. 2. Neutral sentiments are in the blue colour, positive are in cyan colour while negative sentiments are in magenta color.

The two CSV files in the Labelled data folder contain eleven columns. The first column is the numbering of the data in the dataset. The second column is the language of the text followed by the main text which contains the tweets. The fourth column specifies the number of retweets, the fifth column contains the number of replies while the sixth column is the number of quotes of the tweets. The seventh column shows the day the tweet was made, the eighth and ninth column contain the sentiment analysis label while the last two columns contain the sentiment score of the text. The eighth and ninth columns would have been a single column but they were split to ensure that the data is flexible for human interpretation and data visualization. The last two columns were also separated to provide the numerical data that will be used as features y the machine learning models and also for model performance evaluation.

## 4 Experimental Design, Materials and Methods

### 4.1 Data collection, labelling and reprocessing

Data was collected from Twitter using the Twitter API. To have access to the Twitter API, a Twitter developer account was created to generate the API keys and access tokens that were used for authentication, a python program and Tweepy library were used to access the API [2]. The next step was data cleaning and pre-processing which involved importing the Pandas Python library and loading the generated data into a Pandas dataFrame. Pre-processing the imported data included removing duplicate columns, repeated rows, missing values and cleaning the text data to remove special characters and whitespaces [3] and this was achieved by using Pandas and Python's standard libraries like re, string and emoji. The stopwords in the text were removed using the natural language toolkit (nltk) Python library. The stopwords.words (“english”) function provides a list of stopwords in English. Some of the stopwords identified in the dataset are me, at, my, on, the, and, your, yours, about, after, any, should, her, have, over, yourselves, most, any, again and against. The stopwords are words that do not have significant meaning to the text and they were removed so that the focus of the models would be on important words in the text.

### 4.2 Experiment

This study implemented three deep learning approaches which are Convolutional Neural Network, Recurrent Neural Network and a Transformer Model to determine the sentiments of people toward the 2023 Presidential election. Deep learning independently identifies and learn features [2] and it has been applied across a range of fields from recognizing images to detecting biases and establishing rules [3]. Within the broad spectrum of deep learning methods, significant examples are Convolutional Neural Networks, Recurrent Neural Networks, and the Transformer-based framework for opinion mining [4].

### 4.3 Convolutional neural network

The dataset was loaded from a CSV file containing two columns “text” for the input text and “Label” for the sentiment labels. A tokenizer was used to preprocess the text data, converting words into numerical indexes. These numerical sequences were then padded using the pad-sequences function from TensorFlow's Keras preprocessing library to ensure that each input sequence had the same length which is essential for neural network training. Sequences longer than 100 were truncated to 100 elements and those shorter than 100 were padded with zeros. The sentiment labels were transformed into numerical values to match the three sentiment categories assigning a unique number to each model. This step was necessary for the computer to interpret and learn the data. Using TensorFlow's Keras API, a sequential CNN model was built. The process began with an embedding layer that created dense vectors of a predetermined size from the numerical indices. The model employed a standard architecture for feature extraction and downsampling comprising convolutional layers paired with max-pooling layers. The final layer, a dense layer with a softmax activation function provided the probability distribution over the three sentiment classes. For this multi-class classification problem, the model was configured with the Adam optimizer and categorical cross-entropy loss function. To prevent and mitigate overfitting the model was trained on the training set with a validation split.

### 4.4 Recurrent neural network

The dataset was loaded from a CSV file and included text data along with positive, neutral and negative sentiment labels. All prefixes and trailing spaces were removed using Python's strip method during label processing. Tensorflow's Tokenizer was used to tokenize the text data, converting it into a series of integers. To ensure a consistent length for input into neural networks, these sequences were padded. The RNN model was built using the LSTM architecture which is known for effectively handling sequences by preserving long-term dependencies. The model comprised three layers: an LSTM layer to process the sequences, an embedding layer that maps integer indices to dense vectors and a dense output layer with a softmax activation function designed for multi-class classification. The model was constructed using the Adam optimizer and a categorical cross-entropy loss function. It was then trained on the prepared dataset with overfitting monitored and mitigated through validation using a portion of the training data.

### 4.5 Transformer model

The dataset was loaded from a CSV file containing text with negative, positive and neutral sentiment labels. To facilitate model training and performance assessment, the data was split into text and labels arrays and then further divided into training and validation sets. A custom dataset class compatible with PyTorch was created to handle tokenized data. This class encapsulated the tokenized text and labels allowing the training process to effectively process data in batches. The model, training arguments and datasets were instantiated along with the Trainer class. The Trainer object managed the training process including logging, evaluation, and executing training epochs. After training, the model's performance was evaluated using the validation dataset. Comprehensive performance metrics including accuracy, F1 score and AUC (Area Under the Curve) were calculated to assess the model's effectiveness.

## Limitations

Not applicable.

## Ethics Statement

a. Twitter's Terms of Service (TOS) allowed data mining for non-commercial use for Academic Research.
b. The tweets mined for this study are anonymized. Twitter allows for the analysis of Twitter content that does not include personal data.
c. Data redistribution policies were complied with.

## CRediT Author Statement

**Adenike Tosin Odegbile:** Conceptualization, Methodology, Data collecting, Writing, Software and Data Analysis; **Olufemi Moses Oyelami:** Conceptualization, Project administration, Review and Editing.

## Data Availability

Nigeria's 2023 Presidential Election Dataset (Original data) (Mendeley Data)

## References

[1] Tan K.L., Lee C.P., Anbananthen K.S.M., Lim K.M. (2022). RoBERTa-LSTM: a hybrid model for sentiment analysis with transformer and recurrent neural network. IEEE Access.

[2] Sazili S., Ju'im J., Sri I., Riyanto E. (2023). International journal of social science research and review. Int. J. Soc. Sci. Res. Rev..

[3] Braig N., Benz A., Voth S., Breitenbach J., Buettner R. (2023). Machine learning techniques for sentiment analysis of COVID-19-related twitter data. IEEE Access.

[4] Odegbile O. (2024). Adenike; Oyelami, “Nigeria's 2023 presidential election dataset. Mendeley Data.

